# Bilateral changes of TNF-α and IL-10 protein in the lumbar and cervical dorsal root ganglia following a unilateral chronic constriction injury of the sciatic nerve

**DOI:** 10.1186/1742-2094-7-11

**Published:** 2010-02-10

**Authors:** Radim Jančálek, Petr Dubový, Ivana Svíženská, Ilona Klusáková

**Affiliations:** 1 Department of Anatomy, Division of Neuroanatomy, Medical Faculty, Masaryk University, Brno, Czech Republic; 2 Department of Neurosurgery, St. Anne's University Hospital and Medical Faculty, Masaryk University, Brno, Czech Republic

## Abstract

**Background:**

There is a growing body of evidence that unilateral nerve injury induces bilateral response, the mechanism of which is not exactly known. Because cytokines act as crucial signaling molecules for response of peripheral nerves to injury, they may be induced to mediate the reaction in remote structures.

**Methods:**

We studied levels of tumor necrosis factor α (TNF-α) and interleukin 10 (IL-10) proteins using ELISA in the ipsilateral and contralateral lumbar (L4-L5) and cervical (C7-C8) dorsal root ganglia (DRG) from naïve rats, rats operated on to create unilateral chronic constriction injury (CCI) of the sciatic nerve, and sham-operated rats. Withdrawal thresholds for mechanical allodynia and thermal hyperalgesia were measured in the ipsilateral and contralateral hind and forepaws.

**Results:**

The ipsilateral hind paws of all rats operated upon for CCI displayed decreased withdrawal thresholds for mechanical allodynia and thermal hyperalgesia, while no significant behavioral changes were found in the contralateral hind paws and both forepaws. Significantly lower baseline levels of TNF-α and IL-10 protein were measured by ELISA in the lumbar than cervical DRG of naïve rats. Bilateral elevation of TNF-α was induced in both the lumbar and cervical DRG by unilateral CCI of the sciatic nerve for 7 and 14 days, while the level of IL-10 protein was increased bilaterally in the lumbar DRG 1 and 3 days after operation. IL-10 levels declined bilaterally even below baseline level in both cervical and lumbar DRG 7 days from CCI and normalized after 14 days. In contrast to no significant changes in TNF-α, level of IL-10 protein was significantly increased in the ipsilateral lumbar DRG after 3 days and bilaterally in the lumbar DRG after 14 days from sham operation.

**Conclusions:**

The results of our experiments show a bilateral elevation of TNF-α and IL-10 not only in the homonymous DRG but also in the heteronymous DRG unassociated with the injured nerve. This suggests that bilaterally increased levels of TNF-α and IL-10 in DRG following unilateral CCI are linked with general neuroinflammatory reaction of the nervous system to injury rather than only to development and maintenance of neuropathic pain.

## Background

Peripheral neuropathic pain, manifested by spontaneous pain, hyperalgesia and allodynia, arises as a result of various types of nerve damage, e.g., diabetic neuropathy, HIV neuropathy, post-herpetic neuralgia, drug-induced neuropathy and traumatic nerve injury [1,2]. The dorsal root ganglia (DRG) containing the primary sensory neurons play a key role in neuropathic hypersensibility [1,3-5].

Although neuropathic pain usually arises from the area innervated by the damaged nerve, there has been increasing evidence that a peripheral nerve lesion also affects the contralateral non-lesioned side [6]. The majority of studies dealing with peripheral nerve injury observe the contralateral changes at homonymous nerves, but only occasional studies have aimed at reaction of the unaffected heteronymous nerves [7].

Cytokines play a crucial role in the nervous system's reaction to injury. These are signaling proteins that serve as intercellular messengers in immune reaction to injury of the nervous system [8,9]. Experimental studies have provided unequivocal evidence in recent years that the proinflammatory cytokines can induce or facilitate neuropathic pain [10-12]. On the other hand, blockade of proinflammatory cytokines and/or administration of anti-inflammatory cytokines have reduced neuropathic hyperalgesia in animal models [13-16].

Tumor necrosis factor α (TNF-α) is a pleiotropic proinflammatory cytokine that participates in modulation of early degenerative changes during a peripheral nerve injury [17,18]. TNF-α molecules are produced by Schwann and blood derived cells during Wallerian degeneration [19,20] and contribute to both inflammatory [21] and neuropathic hyperalgesia [22]. Following CCI, the level of TNF-α protein is elevated in the distal stump of injured nerve [17] and in the DRG, where increased immunoreactivity has been detected in the satellite glial cells (SGC) and neuronal bodies [23-25].

Interleukin 10 (IL-10) is one of the most important regulators of the immune system. Although IL-10 is known to have many different roles in immune reaction, it is a powerful member of the anti-inflammatory cytokine family, which can suppress many proinflammatory cytokines (e.g., IL-1, TNF-α and IL-6) implicated in neuropathic pain [26-28]. In addition, IL-10 interrupts proinflammatory cytokine signaling by downregulation of proinflammatory cytokine receptor expression [29]. Studies in animal models have shown that IL-10 prevents or reverses many pathological pain states, including pain induced by chronic constriction injury neuropathies [13,30]. The very short biological half-life of the IL-10 protein precludes its direct use for treating neuropathic pain, but it can be delivered using gene therapy techniques and/or in protein compositions with protracted action [31].

Most published studies dealing with the nervous system's reaction to injury are aimed at proinflammatory cytokines. Interest in anti-inflammatory cytokines during pathogenesis of neuropathic pain has increase in recent years. However, data about transitional changes of anti-inflammatory cytokines in DRG following peripheral nerve injury are rare. Therefore, the goal of the present study was to investigate, by means of ELISA analysis, changes of the TNF-α protein as a member of the proinflammatory cytokines and the level of anti-inflammatory IL-10 protein in the ipsilateral and contralateral L4-L5 as well as C7-C8 DRG after both unilateral CCI of the rat sciatic nerve and sham operation.

## Methods

### Animals and surgical procedures

The experiments were carried out on female Wistar rats (n = 42) weighing 240-250 g at the time of surgery. The rats were divided into three principal groups: a group of animals with unilateral chronic constriction injury (CCI) of the sciatic nerve (n = 24), sham-operated animals (n = 12) and a control group of naïve rats (n = 6).

All surgical procedures were performed under aseptic conditions and deep anesthesia induced by a xylazine and ketamine cocktail injected intraperitoneally (xylazine 1.6 mg/kg; ketamine 64 mg/kg). The animals were kept in an animal facility at a temperature of 20-22°C and a natural day-night cycle. Sterilized food and water were available *ad libitum*. All treatment of the animals was in accordance with the European Convention for the Protection of Vertebrate Animals Used for Experimental and Other Scientific Purposes and controlled by the institutional Ethics Committee of Masaryk University in Brno (Czech Republic).

The left sciatic nerve was exposed at mid-thigh and three silk ligatures (Ethicon 3-0) were applied to reduce the nerve diameter by one-third in the rats undergoing CCI. The animals were left to survive for 1 (n = 6), 3 (n = 6), 7 (n = 6) or 14 (n = 6) days. The left sciatic nerve was exposed but no ligature was applied in the groups of sham-operated rats surviving for 3 (n = 6) or 14 (n = 6) days.

### Behavioral tests

Withdrawal thresholds for mechanical allodynia and thermal hyperalgesia were measured in both ipsi- and contralateral hind and forepaws by Dynamic plantar esthesiometer or Plantar test (UGO BASILE), respectively. Rats were first acclimated in clear Plexiglas boxes for 30 min prior to testing. The paws were tested alternately with 5 minute intervals between tests 1 day before operation and 1, 3, 7, and 14 days after operation. Five latency measurements were taken for each paw during each test session. In the case of thermal hyperalgesia, withdrawal time was measured and the intensity radiance (I.R.) was set on the value of 50. Data for mechanical allodynia and thermal hyperalgesia were expressed as mean ± S.E. of withdrawal thresholds in grams and withdrawal latency in seconds, respectively.

### ELISA immunoassay

At the end of the survival times, the animals were sacrificed by carbon dioxide inhalation and the DRG of C7-C8 and L4-L5 segments were exposed bilaterally, removed, and immediately collected into ice-cold PBS (pH 7.4) containing 0.01% Tween-20 and protease inhibitor cocktail (Roche). The DRG removed from rats of each group were collected into four distinct samples: ipsilateral (ipsi-DRG) and contralateral (contra-DRG) cervical and lumbar DRG. The tissue samples were homogenized in ice-cold PBS containing 0.01% Tween-20 and protease inhibitor cocktail, and centrifuged (12,500 g for 12 min) to obtain extract proteins. Blood samples were collected into tubes containing heparin and protease inhibitor cocktail (LaRoche, Switzerland). Plasma was immediately separated by low-speed centrifugation (2500 g for 12 min). Bradford protein assay was used to measure total protein concentration in the tissue supernatant and plasma samples. Commercially available ELISA kits were used for assessing TNF-α (R&D system, MN, USA, sensitivity: 5 pg/ml) and IL-10 (BioSource International, Inc., CA, USA, sensitivity: 5 pg/ml) proteins according to the manufacturers' instructions. Microplates were measured using a SUNRISE Basic microplate reader (Tecan, Salzburg, Austria) and data were standardized as picograms of TNF-α and IL-10 protein to 100 μg of total protein in the supernatant.

### Statistical analyses

Statistical analyses were made with STATISTICA, release 8.0 (StatSoft, Inc., USA). Behavioral data were evaluated using Kruskal-Wallis one-way analysis of variance (ANOVA), and p values less than 0.05 were considered to be significant.

TNF-α and IL-10 protein were measured five times and final data were expressed as mean ± S.D. for each group of animals (naïve, CCI, sham-operated). The values of TNF-α and IL-10 proteins obtained from naïve rats were indicated as the baseline levels. Statistical differences between the naïve and CCI or sham-operated groups for particular periods of survival were tested by the Mann-Whitney U-test, and p values less than 0.05 were considered to be significant.

## Results

### Behavioral tests

**Figure 1:**
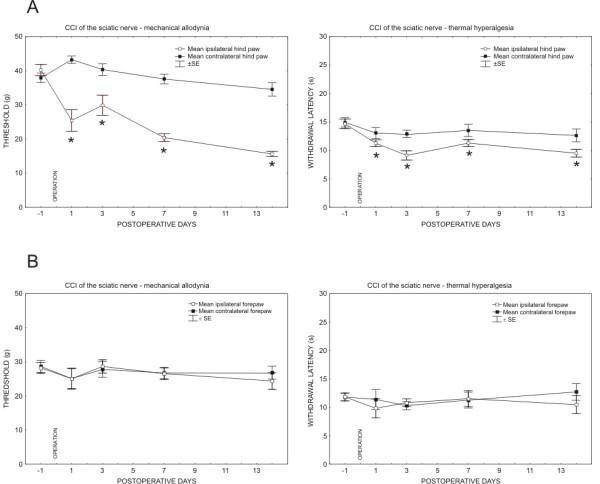
**Results of behavioral tests in the rats operated on to create unilateral CCI of the sciatic nerve**. Progressive development of evoked mechanical allodynia and thermal hyperalgesia was found in the ipsilateral hind paws (A). No significant changes of mechanical allodynia and thermal hyperalgesia were measured in both either the ipsilateral and or contralateral forepaws (B). Data were expressed as mean ± SE of withdrawal thresholds in grams and withdrawal latency in seconds for mechanical allodynia and thermal hyperalgesia, respectively. * indicates statistically significant difference (p < 0.05) when compared with measurement 1 day before operation.

All rats operated on to create CCI of the sciatic nerve displayed a decreased withdrawal threshold for mechanical allodynia and withdrawal latency of thermal hyperalgesia in the ipsilateral hind paws. The contralateral hind paws did not exhibit statistically significant changes of withdrawal threshold for mechanical allodynia when compared with 1 day before operation. Similarly, the forepaws did not display either mechanical allodynia or thermal hyperalgesia up to 14 days (Figure 1). The sham-operated rats did not exhibit mechanical allodynia or thermal hyperalgesia either ipsilaterally or contralaterally to CCI (data not shown).

### Baseline level of TNF-α and IL-10 proteins in the naïve DRG

No significant differences in the levels of TNF-α and IL-10 proteins were obtained between the ipsilateral and contralateral DRG of the same spinal level removed from the naïve rats. ELISA revealed a significantly lower baseline level of TNF-α protein in L4-L5 DRG (2.65 ± 0.93 pg/100 μg) than in C7-C8 DRG (4.65 ± 0.93 pg/100 μg). Similarly, the level of IL-10 protein was lower in L4-L5 DRG (6.26 ± 0.82 pg/100 μg) when compared to C7-C8 DRG (16.33 ± 0.89 pg/100 μg).

### TNF-α protein level following CCI and the sham operation (Figure 2A)

**Figure 2:**
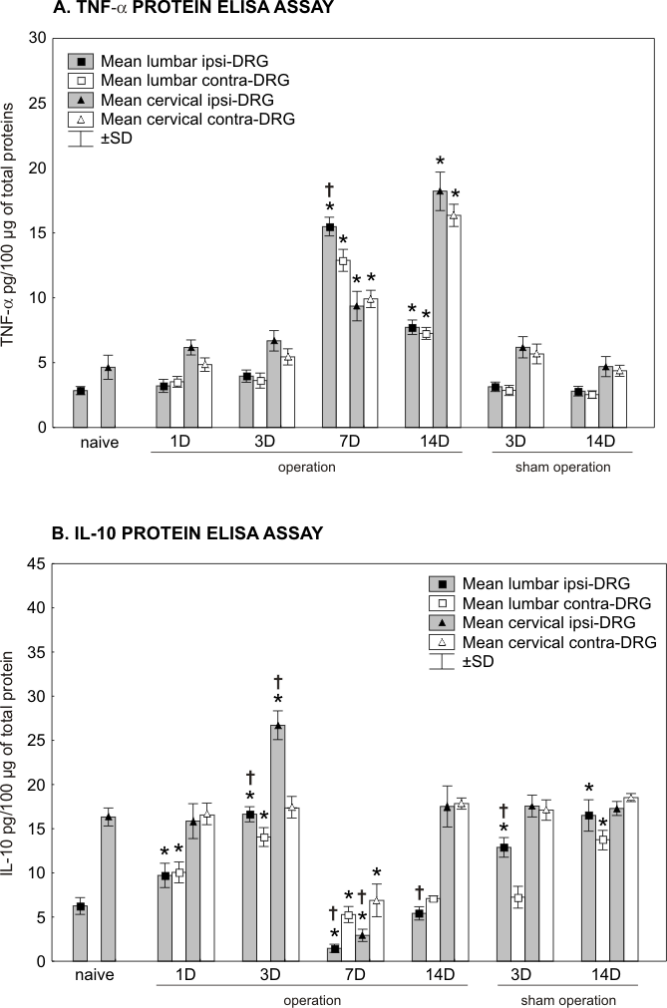
**Results of TNF-α and IL-10 protein levels in DRG**. The levels of TNF-α (A) and IL-10 (B) proteins were measured by ELISA in the ipsilateral (ipsi-DRG) and contralateral (contra-DRG) dorsal root ganglia of lumbar (L4-L5) and cervical (C7-C8) spinal segments removed from naïve rats and rats after unilateral CCI of the sciatic nerve or sham operation. * indicates statistically significant difference (p < 0.05) when compared the protein level of individual times of survival with the baseline level of naïve rats; † indicates statistically significant difference (p < 0.05) when compared the protein level of ipsilateral DRG with contralateral counterparts.

#### L4-L5 DRG

Despite the unilateral CCI of the sciatic nerve, the level of TNF-α protein remained close to the baseline level in L4-L5 DRG of both sides for 1 and 3 days after operation. A bilateral increase of TNF-α protein occurred in L4-L5 DRG after 7 and 14 days of CCI. The peak values were measured after 7 days, when the level of TNF-α protein was more than four times higher in the ipsi- and contralateral L4-L5 DRG than in those from the naïve rats. Compared to at 7 days of survival, the level of TNF-α protein declined bilaterally in L4-L5 DRG removed 14 days after CCI but remained significantly higher than in the naïve DRG. In addition, a significantly higher level (p < 0.05) of TNF-α protein was found in the ipsilateral than contralateral L4-L5 DRG at the period of survival for 7 days. No significant differences were noted between the ipsi- and contralateral L4-5 DRG at 1, 3, and 14 days after CCI.

#### C7-C8 DRG

Surprisingly, unilateral CCI of the sciatic nerve induced significant bilateral changes in the level of TNF-α protein also in C7-C8 DRG. Similarly to L4-L5 DRG, an increase in the level of TNF-α protein was measured bilaterally 7 and 14 days after operation. Unlike L4-L5 DRG, the peak values of TNF-α protein were found bilaterally in C7-C8 DRG after 14 days of CCI, when they were more than four times higher at both sides than in the naïve rats. No significant differences in the levels of TNF-α protein were measured between the ipsilateral and contralateral C7-C8 DRG up to 14 days after unilateral CCI of the sciatic nerve.

#### Sham operation

The levels of TNF-α protein remained without significant changes in both L4-L5 and C7-C8 DRG at 3 and 14 days after the sham operation.

### IL-10 protein level following CCI and sham operation (Figure 2B)

#### L4-L5 DRG

Unilateral CCI of the sciatic nerve induced a significant bilateral increase of IL-10 protein level in L4-L5 DRG as early as 1 day from the operation. The level of IL-10 protein peaked bilaterally in L4-L5 DRG after 3 days of CCI. A subsequent sharp decline of IL-10 protein below the baseline level of the naïve DRG was found in L4-L5 DRG of both sides, but more so in the ipsilateral ones. The baseline level of IL-10 protein was achieved bilaterally 14 days from the operation. With 1 day of CCI being the exception, significant differences in IL-10 protein level were detected between the ipsilateral and contralateral L4-L5 DRG at 3, 7, and 14 days from operation.

#### C7-C8 DRG

Significant changes of IL-10 protein levels were also induced in C7-C8 DRG by the unilateral CCI of the sciatic nerve. While no changes were present 1 day from CCI, an increased level of IL-10 protein was measured in the ipsilateral C7-C8 DRG at day 3. As with L4-L5 DRG, a bilateral decrease below the baseline level of the naïve DRG was observed after 7 days. More distinct diminution was found in DRG of the ipsilateral side. The level of IL-10 protein was normalized 14 days from the operation, at which time no significant differences of IL-10 protein were noted between the ipsilateral and contralateral C7-C8 DRG.

#### Sham operation

The sham operation for 3 days induced a significant increase of IL-10 protein in the ipsilateral L4-L5 DRG. A bilateral increase of IL-10 protein occurred in L4-L5 DRG after 14 days, when the protein level was similar to that from 3 days after CCI. No changes of the IL-10 protein levels were found in C7-C8 DRG of the sham-operated rats after 3 and 14 days.

### TNF-α and IL-10 protein levels in plasma

The levels of TNF-α and IL-10 proteins were also measured in the plasma of all three principal groups of rats. The baseline levels of TNF-α and IL-10 proteins in plasma of the naïve rats averaged 0.12 ± 0.01 pg/100 μg and 0.10 ± 0.02 pg/100 μg, respectively. No significant changes were found in TNF-α and IL-10 protein levels in the plasma of CCI or sham-operated rats when compared with the baseline level of the naïve animals.

## Discussion

There is unequivocal evidence that pro-inflammatory cytokines mediate cellular and/or molecular changes in DRG manifested by hyperexcitability of the primary sensory neurons, and thus they contribute to both induction and maintenance of neuropathic pain [20,24,32,33].

Chronic constriction injury (CCI) of a peripheral nerve by chromic gut [34] is a widely used experimental model with characteristic signs of neuropathic pain. The original CCI model of neuropathic pain is not suitable for distinguishing the local inflammatory reaction induced by a thread material and neuroinflammation as a manifestation of Wallerian degeneration of injured axons [35]. In our experiments, therefore, we applied the sciatic nerve ligation using a 3-0 sterilized thread (Ethicon) under aseptic conditions to study levels of TNF-α and IL-10 proteins in DRG. The changes of TNF-α and IL-10 proteins in the ipsi- and contralateral L4-L5 DRG as well as C7-C8 DRG were therefore largely induced by traumatic nerve injury and accompanying neuroinflammatory response.

There is conclusive evidence that TNF-α is involved in ectopic changes of DRG neurons inducing neuropathic pain [36,37]. Immunofluorescence staining has revealed that neuronal bodies, their satellite glial cells and ED-1+ macrophages infiltrating ipsilateral DRG are main cellular sources of TNF-α [20,38,39]. However, the number of ED-1+ macrophages in DRG contralateral to nerve injury was significantly increased no sooner than 4 weeks following CCI [25] and principally was not detected in DRG heteronymous to injured nerve. Therefore, neuronal bodies and their satellite glial cells are probably responsible for elevation of TNF-α protein in the contralateral L4-L5 DRG and bilateral C7-C8 DRG following unilateral CCI of the sciatic nerve.

Anti-inflammatory IL-10 is produced by a variety of immune cell types, including cells of the monocyte/macrophage lineage [40,41]. It has been also proven that IL-10 is expressed predominantly in small-sized DRG neurons [38]. Exogenous administration of anti-inflammatory cytokine IL-10 impeded development of prodynorphin-induced allodynia [42,43] and inhibited endotoxin-induced hyperalgesia through downregulation of proinflammatory cytokines [44,45]. In contrast, results of experiments with IL-10 knockout (IL-10-/-) mice or normal (IL-10 +/+) mice treated with IL-10 antibody indicate that endogenous IL-10 effectively increases nociception [46].

Based on ELISA analysis, we found a higher baseline level of TNF-α and IL-10 proteins in naïve DRG of the cervical than lumbar spinal segments. The differences could be caused by a presence of diverse populations of the primary sensory neurons in various spinal cord levels [47]. Moreover, natural heterogeneity in composition alongside the neuraxis is also documented by a rostro-caudal gradient of amino acid neurotransmitters in cerebrospinal fluid [48].

A bilateral elevation of TNF-α protein in both the lumbar and cervical DRG was induced by unilateral CCI of the sciatic nerve for 7 and 14 days, while the level of IL-10 protein was increased bilaterally in the lumbar DRG 1 and 3 days after CCI and in the ipsilateral cervical DRG 3 days from operation. Generally, these results indicate certain relationships between transient and early rise of anti-inflammatory IL-10 and later increase of proinflammatory TNF-α. IL-10 influences the proinflammatory cytokines in various ways and prevents a transition of the physiologic inflammatory reaction to a pathologic state that may result in neuropathic pain. IL-10's action is based on a selective inhibition of the synthesis and release of proinflammatory cytokines [49]. Surprisingly, IL-10 protein levels showed an extreme bilateral drop in both the cervical and lumbar DRG 7 days from CCI. A transient decrease of IL-10 protein level in DRG following CCI may explain why various treatments increasing local IL-10 concentration may be beneficial in neuropathic pain states [30,50].

Cytokine networks are generally extremely intricate with many cytokine-cytokine interactions. The nature of cytokine-cytokine interactions is such that their net output could be additive, synergistic, or antagonistic [51]. Peripheral nerve injury induces a neuroinflammatory reaction with expression of essentially the same pattern of cytokines in a highly ordered fashion [52]. The delicate balance between proinflammatory and anti-inflammatory cytokines is pivotal in formation of conditions suitable for successful nerve repair [53]. On the other hand, dysregulation in neuroinflammatory response to nerve injury as well as exogenous application of proinflammatory cytokine (e.g., TNF-α, IL-1β, and IL-6) leads to imbalance of the endogenous cytokine network that results in a variety of disease states such as neuropathic pain [54]. Although the altered levels of TNF-α and IL-10 proteins in DRG associated with the damaged nerve may indicate a relation of cytokines to proved hypersensitivity in ipsilateral hind paws, the results of contralateral DRG as well as DRG non-associated with injured nerve also argue for other role(s) of cytokines in reaction of the nervous system to injury. Therefore, the results indicate that the bilateral increase in the level of TNF-α and IL-10 proteins in DRG following unilateral CCI is probably linked with a general neuroinflammatory reaction of the nervous system to injury rather than its being only a condition for development and maintenance of neuropathic pain.

### Changes of TNF-α and IL-10 protein level in the contralateral DRG

There is a growing body of evidence that a unilateral nerve injury induces contralateral changes. The effect of the unilateral peripheral nerve lesion on contralateral non-lesioned structures was reviewed by Koltzenburg and colleagues [6]. Recently, further studies have verified the concept of contralateral reaction to unilateral nerve damage [7,39,55]. It is generally accepted that responses to contralateral injuries are usually qualitatively similar but smaller in magnitude and have a briefer time course compared to ipsilateral changes [6]. However, our results for TNF-α and IL-10 proteins analyzed by ELISA demonstrate an effect of the unilateral nerve injury on the contralateral DRG that is mostly of the same magnitude as that on the ipsilateral DRG.

### Changes of TNF-α and IL-10 protein levels in DRG heteronymous to injured nerve

According to previously published findings, a peripheral nerve injury induces contralateral changes limited to the homonymous nerves. Contralateral response of cytokines after a unilateral sciatic nerve injury was restricted to the homonymous opposite sciatic nerve but spared the femoral nerve [7]. Our results reveal that unilateral CCI of the sciatic nerve induced bilateral changes of TNF-α and IL-10 proteins not only in homonymous L4-L5 DRG but also in C7-C8 DRG that are heteronymous to the injured sciatic nerve. Similar quantitative changes of cytokine proteins found in both the ipsilateral and contralateral DRG of lumbar and cervical levels following unilateral CCI suggest the same principal regulation of TNF-α and IL-10 protein levels. However, the mechanisms of signaling for alteration of cytokines in the DRG unassociated with injured nerve have not yet been clearly elucidated. Generally, it seems that extension of DRG reaction into heteronymous levels can be principally mediated by neuronal or non-neuronal signaling.

The first type of signaling of physiological imbalance induced by unilateral nerve injury could be transferred to homonymous contralateral or heteronymous DRG of both sides by neuronal activity alongside neuronal pathways, e.g., through interneurons at the spinal cord segment or supraspinal levels [56-58]. It has been demonstrated that rats with unilateral nerve injury displayed increased neuronal activity in wide dynamic range (WDR) neurons on the contralateral side of the spinal cord. A majority of WDR neurons responded to contralateral noxious stimulation in CCI rats as compared to the intact rats [57]. Moreover, there are long ascending propriospinal systems linking the lumbar and cervical spinal cord segments. The so-called long ascending propriospinal neurons are defined as interneurons whose somata are located in the lumbar spinal cord segments and whose axons terminate in the cervical segments. They are in an anatomically appropriate position to participate in coordinating movements of hind limbs and forelimbs [59,60].

The present findings of bilateral changes in TNF-α and IL-10 protein levels in DRG homonymous and heteronymous with damaged nerve indicate that systemic signaling (e.g., via the bloodstream) cannot be excluded. Wallerian degeneration distal to the sciatic nerve ligature results in interruption of the blood-nerve barrier [61,62], thus allowing diffusion of signaling molecules produced by the Schwann and immune cells into blood flow [63,64]. An absence of blood-nerve barrier in the intact DRG [65-67] supports a possibility for diffusion of circulating signal molecules into the microenvironment of the DRG not associated with injured nerve. Several candidate molecules have been suggested for signaling from damaged nerve, including, for example, ATP, glutamate, complement or damaged nerve-derived molecules and toll-like receptors (TLRs) [7,68-71]. The blood flow is probably another route for transportation of the signaling molecules from injured nerve stump to the proximity of afferent neurons in DRG not only associated but also unassociated with the damaged nerve.

We also cannot exclude a dynamic interplay among the hypothalamic-pituitary-adrenal (HPA) axis, stress, and neuroimmune reaction to nerve injury as they relate to the development and maintenance of neuropathic pain [72,73].

### Changes of TNF-α and IL-10 protein levels in DRG of sham-operated rats

The fact that there were no changes of TNF-α protein levels in DRG removed from sham-operated rats indicates that the surgical approach did not itself contribute to elevation of this proinflammatory cytokine up to 14 days. In contrast to TNF-α protein, a significant elevation of IL-10 protein in DRG from sham-operated rats may reflect an injury to a small amount of afferent nerve fibers during the surgical approach. This possibility is supported by later bilateral elevation of IL-10 in the lumbar DRG (14 days from sham operation) while CCI induced bilateral increase already after 1 day. In contrast, the fact that there were no changes of TNF-α in DRG of sham-operated rats after even 14 days could be explained by belated regulation of TNF-α protein due to tissue damage and as illustrated by the first significant alternation's occurring as late as 7 days from CCI.

## Conclusions

In conclusion, a peripheral nerve injury results in a shift of DRG neuronal functions from normal maintenance and neurotransmission toward survival and regeneration status. Regeneration of nervous tissue is connected with release of many biologically active substances, including cytokines, that may induce neuropathic pain (for a review, see Woolf, 2004)[74]. Bilateral changes of TNF-α and IL-10 protein in both the lumbar and cervical DRG following unilateral CCI were related to significant decrease of thresholds for mechanical allodynia and thermal hyperalgesia only in ipsilateral hind paws. Therefore, our results indicate that bilateral increase in the level of TNF-α and IL-10 in DRG following unilateral CCI is linked with general neuroinflammatory reaction of the nervous system to injury rather than its being only a condition for development and maintenance of neuropathic pain. These findings may have implications for future study design and therapeutic approaches to neuropathic pain.

## Competing interests

The authors declare that they have no competing interests.

## Authors' contributions

RJ participated in the design of the study, evaluation and statistical analyses of the results, and drafting of the manuscript. PD participated in the design of the study, evaluation and statistical analyses of the results, and drafting of the manuscript.

IS participated in surgical procedures. IK participated in surgical procedures.

All authors read and approved the final manuscript.
